# Personalized prediction of mode of cardiac death in heart failure using supervised machine learning in the context of cardiac innervation imaging

**DOI:** 10.1007/s12350-020-02215-z

**Published:** 2020-06-17

**Authors:** Rudolf A. Werner, Thorsten Derlin, Frank M. Bengel

**Affiliations:** Department of Nuclear Medicine, Hannover Medical School, Hannover, Germany

In recent years, the use of artificial intelligence (AI) has attracted interest for numerous medical imaging tasks.^1^ Striving to identify comparable or even identical patterns in large datasets, AI and its subdomain machine learning (ML) may hold promise to address the urgent need of analyzing complex interactions between multiple data points, ultimately allowing to identify high-risk individuals prone to major clinical events, to speed up clinical trials by selecting the most suitable candidates, or to provide therapeutic guidance by offering personalized treatment.^2^

In general, ML is based on the concept of probability. Based on previously fed data, ML makes assumptions with an increasing degree of certainty. Implementation of feedback loops allows for learning, i.e., after being told that the initially chosen decision was incorrect and will not lead to a favorable outcome, the procedure will be modified and the task will be re-assessed by the ML algorithm until the desired expectations have been met.^2^,^3^

In a manner similar to myocardial perfusion imaging which had a pioneering role in nuclear medicine in the early 1970s,^4^,^5^ nuclear cardiology is again at the forefront of innovation in molecular imaging by applying supervised ML for outcome prediction to different clinical scenarios.^6^,^7^ In the present issue of the *Journal of Nuclear Cardiology*, *Nakajima* et al. expanded the use of ML to the field of cardiac innervation imaging by incorporating the cardiac nerve radiotracer [^123^I]-metaiodobenzylguanidine ([^123^I]-mIBG) in an AI-based risk prediction model for segregating between low-risk vs high-risk patients potentially experiencing end-stage heart failure (HF) death or life-threatening arrhythmic events (ArE). Given the difficult management of these high-risk individuals, which includes cardioverter defibrillators and resynchronization therapy, reliable risk stratification is needed. To tackle this challenging task, the authors established an ML-based approach in the context of [^123^I]-mIBG imaging, which could identify subjects at risk of fatal ArE and end-stage HF death. Interestingly, the ML-derived risk prediction model showed that risk for ArE was markedly elevated at the intermediate range of SPECT-derived heart-to-mediastinum ratio (HMR) in younger subjects with less severe HF. This finding can be explained by increased arrhythmogenicity of denervated but viable myocardium, in particular as the imbalance between preserved cardiac perfusion and impaired cardiac nerve integrity seems to be an important driver of serious arrhythmias.^8^ Moreover, in the present paper, *Nakajima* et al. could also verify the consistent correlation of HF-related increase of death with decreased cardiac [^123^I]-mIBG uptake in the elder subjects with worse New York Heart Association (NYHA) functional class and higher frequency of comorbidities. These observations are in line with results of the *ESC-Failure Pilot study*, also reporting on increased prevalence of pump failure death in older patients with severe symptoms and worse NYHA status.^9^

Taken together, the present paper demonstrates that ML-based re-assessment of [^123^I]-mIBG-derived HMR may be of relevance, as such an approach can not only predict one particular type of cardiac death mode in a restricted patient population, but is applicable to a broad range of various clinical settings, including congestive HF-related terminal endpoints in the elderly vs ArE-driven cause of death in younger subjects. These considerations may open avenues for a more sophisticated approach of applying ML to cardiac nerve PET radiotracers, as more detailed quantitative information derived by PET may hold promise to risk stratify between other high-risk individuals prone to major cardiovascular events. Established ^11^C- and innovative ^18^F-labeled PET radiotracers for cardiac nerve integrity are available^10^ and the improved spatiotemporal resolution of PET technology may allow for an in-depth assessment of sympathetic innervation of the myocardium, including quantification of regional heterogeneity such as the viable border zone after myocardial infarction.^11^ Compared to a global assessment of cardiac innervation as derived by SPECT-based HMR, the additionally gained quantitative data by PET could even further improve the herein presented risk estimations using ML-based classifiers.^12^ Moreover, PET enables for a non-invasive whole-body read-out and thus, information from other organs in the field of view could also be considered and fed into ML-based approaches for response prediction.^4^,^13^,^14^
